# Spatial Disparities of COVID-19 Cases and Fatalities in United States Counties

**DOI:** 10.3390/ijerph18168259

**Published:** 2021-08-04

**Authors:** Sarah L. Jackson, Sahar Derakhshan, Leah Blackwood, Logan Lee, Qian Huang, Margot Habets, Susan L. Cutter

**Affiliations:** Department of Geography, Hazards & Vulnerability Research Institute, University of South Carolina, Columbia, SC 29208, USA; saharder@email.sc.edu (S.D.); lmmoore@email.sc.edu (L.B.); logan.lee@westpoint.edu (L.L.); qh1@email.sc.edu (Q.H.); mhabets@email.sc.edu (M.H.); scutter@mailbox.sc.edu (S.L.C.)

**Keywords:** COVID-19, urban, rural, social vulnerability, resilience, mitigation, recovery, GIS

## Abstract

This paper examines the spatial and temporal trends in county-level COVID-19 cases and fatalities in the United States during the first year of the pandemic (January 2020–January 2021). Statistical and geospatial analyses highlight greater impacts in the Great Plains, Southwestern and Southern regions based on cases and fatalities per 100,000 population. Significant case and fatality spatial clusters were most prevalent between November 2020 and January 2021. Distinct urban–rural differences in COVID-19 experiences uncovered higher rural cases and fatalities per 100,000 population and fewer government mitigation actions enacted in rural counties. High levels of social vulnerability and the absence of mitigation policies were significantly associated with higher fatalities, while existing community resilience had more influential spatial explanatory power. Using differences in percentage unemployment changes between 2019 and 2020 as a proxy for pre-emergent recovery revealed urban counties were hit harder in the early months of the pandemic, corresponding with imposed government mitigation policies. This longitudinal, place-based study confirms some early urban–rural patterns initially observed in the pandemic, as well as the disparate COVID-19 experiences among socially vulnerable populations. The results are critical in identifying geographic disparities in COVID-19 exposures and outcomes and providing the evidentiary basis for targeting pandemic recovery.

## 1. Introduction

Local population dynamics and sociodemographic characteristics have proven influential on the prevalence of coronavirus disease 2019 (COVID-19) impacts and transmissibility. However, early mitigation measures did not account for place-based differences in exposure and outcomes at comparative and more localized spatial scales [1]. Unprecedented United States government mitigative policies led to drastic unemployment increases to prevent the spread of COVID-19, which did not occur equally across the country [2]. Initial COVID-19 cases and fatalities in the US first appeared in densely populated urban centers [3], before spreading to rural communities throughout the country [4]. Rural populations continually face a unique set of challenges relating to unequal access to healthcare and a higher prevalence of underlying health conditions, thus placing them at higher risk of negative COVID-19 outcomes [5]. Underlying disparities in social vulnerability, community resilience and COVID-19 mitigation measures among US communities and urban–rural places must then influence COVID-19 exposure and outcomes, but how?

Existing COVID-19 research has not integrated the full suite of social vulnerability measurements along with multiple societal elements of place that could explain patterns of COVID-19 cases and fatalities within local geographic contexts. Additionally, spatial analyses of initial community recovery based on unemployment across the US lack investigation, despite the vastly disproportionate impact of the pandemic mitigation orders on the job market. Applying measurements of social vulnerability, community resilience, government mitigation efforts and unemployment changes, this place-based geographical investigation examines spatial and temporal differences in COVID-19 exposures (cases) and outcomes (fatalities) at the county level, using statistical and geo-analytical methods. Our results identify how combined contextual correlates explain the geographic distribution of cases/fatalities and initial economic recovery throughout the first year of the pandemic (January 2020–January 2021). Three questions guide this analysis:

1. What is the variability in county spatial and temporal patterns of COVID-19 cases and fatalities?
2. What is the relationship between COVID-19 cases and fatalities, social vulnerability, community resilience and government mitigation measures and does this vary based on location (urban–rural)?
3. Do differential spatial patterns of pre-emergent recovery exist using changes in unemployment as a proxy indicator?

## 2. Background

From the outset of the pandemic, research and popular concern focused on the disproportionate impact on minority, low-income and elderly populations [6,7,8]. Only recently has there been a focus on place, or what Perry et al. [9] term the precarity of place, in addition to urban–rural influences on COVID-19 experiences [10]. Research has mostly examined the geography of COVID-19 risk based on individual-level factors (e.g., social determinants of health) or community-based elements (e.g., resilience, social vulnerability, or public health mitigation measures) over a short study period or wave of the pandemic [11,12], or in specific sub-national areas [9,13,14]. Thus far, there has not been a longer-term (year-long) study for the US examining the spatial and temporal county-level variability in COVID-19 cases and fatalities that considers several influential elements of social context or measurements of pre-emergent economic recovery. 

### 2.1. Socioeconomic and Spatial Disparities of the COVID-19 Pandemic

The social determinants of health and social vulnerability to environmental hazards are crucial elements to consider in evaluating the spatial and temporal dynamics of the COVID-19 pandemic. The social determinants of health are the non-medical factors (e.g., income, wealth, education, race and ethnicity, geographic location and gender) that can shape an individual’s health in formative ways [15,16]. Social vulnerability is widely studied in geography, anthropology, economics, public health and psychology to identify inequalities and sub-populations that are more at risk to hazard events [17,18]. While definitions of social vulnerability may vary by discipline, it is widely agreed that not all people and places cope with and adapt to hazards equally, whether from institutional barriers, human and social capacities, or the physical environment [19,20,21]. In the context of COVID-19, minority status and crowded housing conditions have correlated with case rates, as well as overall levels of social vulnerability [11,22]. Spatial variability in COVID-19 exposures and outcomes among different demographic and socioeconomic groups highlights the role of race and non-English speakers in elevated case counts, as well as age and disability associated with elevated fatalities [5,12,23]. African Americans have a 2.7 times greater chance of being hospitalized for COVID-19 than white patients [24], as well as an increased likelihood of testing positive for COVID-19 [25,26]. Public health inequities arise from socioeconomic disadvantages [27] and COVID-19 is no exception.

In addition to examining how the spatial variance of socioeconomic variables impact the spatial distribution of COVID-19 cases and fatalities, recent research has found a geographic pandemic divide. Regionally, the Southeast, Southwest and New England experienced relatively more COVID-19 cases than other US regions [23]. These and other regional patterns [28,29] may not hold over a longer study time-period. Urban–rural disparities explored in earlier research found increased mortality rates [30] and higher prevalence rates of COVID-19 infection in urban counties [12]. Another study found higher standardized cases and fatalities in rural counties in just one US state [13], while others found severe negative impacts of the pandemic on rural unemployment and economic wellbeing [10]. Not only do rural residents have higher positive COVD-19 testing rates [26] and fewer hospitalization resources available for affected patients [31,32], but locational disparities exist in testing access and pandemic messaging in rural locations [33]. 

### 2.2. Community Resilience to Hazards

Resilience addresses people and places’ ability to withstand the adverse effects of hazards, as well as their ability to recover from and adapt to hazards [34] at multiple scales (e.g., individual, family and community). Studies have represented the multidimensional aspect of community resilience using domains signifying social, physical, community, individual, economic, institutional, infrastructure and/or ecological characteristics [35,36]. Common variables used to measure resilience include community-level variables, such as wealth, participation in civic and religious organizations, redundancy of critical infrastructure and hazard mitigation planning [35,37]. Community resilience and social vulnerability are related, but they are not the opposite or inverse of each other, with empirical studies showing a negative relationship with moderate strength between vulnerability and resilience [38,39,40,41]. 

### 2.3. COVID-19 Mitigation and Pre-Emergent Recovery

Research confirms that mobility restrictions (i.e., work-from-home, shelter-in-place, or stay-at-home orders), physical distancing and mask mandates lower COVID-19 confirmed cases and fatalities [42,43,44]. Early analyses of the COVID-19 outbreak in Wuhan, China, support the significance of limiting all travel to effectively control the spread during public health response planning [45]. In the US, mitigative efforts via work-from-home orders lessened the spread of COVID-19 cases between some communities [46]. Yet, Berry et al. [47] found little evidence to support that shelter-in-place orders affected disease spread or fatalities. Comparisons of voluntary mitigative actions versus mandatory stay-at-home orders found that, in counties with existing voluntary behaviors, the mandates simply accelerated compliance [48]. However, not all sociodemographic groups have the social or economic capital available to partake in voluntary mitigative action and can thus suffer worse economic burdens from such imposed mitigation orders [49]. Additionally, some research has shown the relative effectiveness of government orders on mobility reductions and individuals’ voluntary mitigation decisions based on political partisanship [50,51,52].

Recovery is “both a social process with specific short and longer-term outcomes and a physical process of replacing the damaged built environment (or reconstructing it)…” [53] (p. 5). Economic recovery as part of the social process requires not only containing the spread of the virus and developing the vaccine, but also making and implementing policies that protect people’s livelihoods, minimize financial suffering and place the economy in a better position for a faster resurgence [54]. In response to the COVID-19 pandemic, many countries have launched economic recovery programs to mitigate unemployment and stabilize core industries [55]. The US labor market is undergoing tremendous stress because of the COVID-19 outbreak and mitigation efforts [56], with many individuals becoming unemployed and losing health insurance coverage as a result [57]. A survey conducted in the early stage of the pandemic showed that 43% of small businesses temporarily closed and that employment had fallen by 40% [58]. Those impacted most heavily from job loss include some highly socially vulnerable populations of low-income individuals and/or racial/ethnic minorities working in employment sectors most affected by mitigation policies [2,49,59]. By April 2020, all 50 US states began easing mobility or business closure restrictions to revive the economy [60].

## 3. Research Design and Methods

Counties are the primary spatial unit for operational levels of emergency management and public health statistics in the US, thus the appropriate unit of analysis. This study includes 3140 counties or county-equivalent places in all 50 states and the District of Columbia. Two low population county-equivalents in Alaska, Yakutat City and Borough and Hoonah-Angoon Census Area, were not included in the analysis due to missing COVID-19 reports in the dataset. 

### 3.1. Data

Table 1 summarizes all spatial data inputs. Listed below are detailed explanations of the input data, spatial caveats and initial data manipulations.

#### 3.1.1. COVID-19 Cases and Fatalities

Publicly available COVID-19 daily cumulative case and fatality counts were downloaded from The New York Times GitHUB [61] on 31 January 2021. The dataset includes counts for all US counties and was compiled based on reports from state and local governments, as well as local health departments, beginning with the first reported case in Washington State on 21 January 2020. Data included both confirmed cases (positive SARS-CoV-2 RNA laboratory test) and probable cases (based on criteria for symptoms, exposure and antibody testing). Confirmed deaths listed COVID-19 as the cause of death, while probable deaths were those with COVID-19 listed on the death certificate, but without a positive laboratory test. All counts for cases and deaths (confirmed and probable) used patients’ county of residence. Daily case and fatality reports occasionally lacked an accurate county identifier for a patient, leading to some data recorded as “Unknown”. In some instances, the availability of more accurate information enabled locational corrections to these unknown cases/fatalities, while, in others, they were not corrected [61]. In our analysis, 22 states had geographically unassigned cases and/or fatalities at the end of the study period, on 30 January 2021. Rather than eliminate these data, we proportionally distributed the data among counties based on existing ratios of county cases/fatalities in each state, by epidemiological (epi) week.

Calculated cumulative totals of standardized cases and fatalities (total cases or fatalities per 100,000 population) used the American Community Survey (ACS) 2019 5-year population estimates [62]. Daily case and fatality totals by county were aggregated per epi week to analyze temporal patterns. 

A few key geographic issues in the data were fixed to create a consistent geo-referenced dataset for this investigation. In New York City, the dataset reported all cases as one geographic entity, not five separately representing the counties (Boroughs) in the city. Case and fatality values for the five New York City boroughs came from the New York City Department of Health GitHUB [72] and the proportions of cases/fatalities for each borough per epi week were calculated and applied to distribute The New York Times data into each borough. In Kansas City, Missouri, the dataset reported the city’s cases and fatalities separately from the four counties that overlap the city (Cass, Clay, Jackson and Platte) and the county values did not include Kansas City cases or fatalities. A similar instance occurred in Joplin, Missouri, after 25 June 2020, when cases and fatalities reported for the city became separately counted and not included in Jasper and Newton county totals. An areal-weighted approach to attribute cases/fatalities to the surrounding counties of Kansas City and Joplin helped address this spatial data issue. While more sophisticated techniques exist to estimate populations in census boundaries that do not assume a homogenous distribution of the data, areal weighting is a relatively straightforward method of assigning data based on the area of the city intersecting county boundaries. Other studies using The New York Times dataset did not address these spatial anomalies in the data for those cities, indicating they were simply excluded from analyses [73,74,75].

#### 3.1.2. Social Vulnerability and Resilience

The Social Vulnerability Index (SVI) from the Centers for Disease Control and Prevention (CDC) [76] and the Social Vulnerability Index (SoVI^®^) from the Hazards and Vulnerability Research Institute at the University of South Carolina [63] are the two most often cited quantitative measures of social vulnerability [77]. Although named the same, there are key differences in their composition and formulation. SoVI^®^ utilizes more sociodemographic and socioeconomic variables as proxies for social vulnerability than SVI. SoVI^®^ applies an inductive method of grouping variables that are highly correlated into factors of vulnerability, while SVI uses a hierarchical approach [78]. SoVI^®^ includes important place and health-based indicators of vulnerability that are excluded from SVI, such as the number of hospitals per capita and the percent of the population without health insurance, as well as economic indicators of vulnerability (e.g., percent employment in the volatile and seasonal service sector industry). Our research employs SoVI^®^ as the measure of social vulnerability due to these key differences and because SoVI^®^ has proven more reliable in studies validating the indices using disaster outcome measures [77]. Key variables inputted into SoVI^®^ include (but are not limited to) age, wealth, race, ethnicity and education level. SoVI^®^ values are relative and range from a low of −9.01 in Loudoun County, Virginia (least vulnerable) to a high value of 15.52 in Kusilvak Census Area, Alaska (most vulnerable).

The Baseline Resilience Indicators for Communities (BRIC) applied here is a well-known quantitative measure for community resilience measurement. BRIC uses 49 variables that are separated into six capitals of resilience (social, economic, institutional, housing/infrastructure, environmental and community). Standardized variables have values ranging from 0 to 1, which are then averaged for each capital. The sum of the capitals has a theoretical range of 0–6 for each county, with higher scores representing more resilience and lower scores less resilience [64].

#### 3.1.3. County and State COVID-19 Mitigation Efforts

To measure the COVID-19 mitigation efforts at both the county and state level, we analyzed data from four sources (Table 1). A binary system was created with zero (0) indicating the absence of the mandate and one (1) indicating the presence of a mandate (i.e., mask, emergency declaration, stay-at-home policy/order, business closures) for each of the three county level datasets. To avoid double-counting, triangulation among the three datasets produced a single value (0 or 1) for each county for all four mitigation measures. Scores ranged from 0–4 for county-ordered mitigation. Another mitigation dataset had state-level actions only (Table 1) and included emergency declarations, shelter-in-place orders, business closures and mask mandates. In addition, using a binary system, if there was a statewide mandate for any of the mitigation measures, all counties in the state received a score of one for the presence of that mandate and zero if there was no mandate. As with the county-specific mandates, scores ranged from 0 to 4 for statewide measures. We assessed the impact of mitigation methods for county-specific and statewide separately and in combination (adding the two scores so the hypothetical range was 0–8). Eighteen counties elected to go against their state-level mask policy and opt-out of the mandate, so these counties received a value of 0 instead of 1 for mask mandate in the dataset.

#### 3.1.4. Pre-Emergent Economic Recovery

Even though COVID-19 continues to spread, an initial economic recovery is underway. Communities have begun the process of a return to normalcy by loosening restrictions on businesses as vaccinations increased and the federal government unveiled massive stimulus packages to encourage consumer spending and help those that have lost their jobs. To measure and identify where recovery was the strongest during our study period, the unemployment rate from the previous year (2019) was compared to the year of the pandemic (2020), then examined in monthly intervals. This enabled a relative comparison of places where unemployment rose or fell creating a proxy level of recovery in those places at the end of January 2021. Unemployment data were derived from the US Bureau of Labor Statistics [69] for each month from January 2019 to January 2021 and for each county in the U.S, except for Kalawao County, Hawai’i. The change in the unemployment rate from a year before the pandemic (2019) to the pandemic year (2020) was computed in monthly intervals; then, a summary measure of percent change in unemployment for the entire study period for each county was calculated. To calculate this measure, the 2019 monthly unemployment rate was averaged for the year, then subtracted from the value for 2020 calculated the same way, in order to provide the unemployment rate percent change from 2019 to 2020. We recognize that recovery is ongoing but want to test the spatial patterns of economic recovery with the distribution of cases and fatalities of COVID-19.

#### 3.1.5. Urban–Rural Delineations of US Counties

The spatial delineations of urban–rural areas in the US vary amongst a few federal government agencies, including the CDC’s National Center for Health Statistics (NCHS) [70] and the US Department of Agriculture (USDA) [71]. One of the main US urban–rural federal classification schemes currently employed at county level is the NCHS 2013 Urban–Rural Classification Scheme, which assigns one of six urbanization levels to US counties, with 1–4 representing metro counties and 5–6 being non-metro [70]. Another scheme is the nine-level USDA 2013 Rural-Urban Continuum Codes (RUCCs), with 1–3 assigned to metro counties and 4–9 to non-metro counties [71]. Creating a binary non-metro/rural (0) and metro/urban (1) categorization for analysis using either the NCHS or USDA schema results in the same designations for each US county.

### 3.2. Analytical Approach

A range of statistical methods informed our analysis, including descriptive and inferential statistics, geospatial statistics and GIS analytics. Microsoft Excel 2016 was used to manage the tabular datasets prior to statistical and spatial analyses. The SPSS 27.0 software (IBM Corp., Armonk, NY, USA) was used to examine statistical associations via correlation and difference of means testing between standardized cases/fatalities, location (urban/rural), statewide mitigation, county mitigation, unemployment percent change, social vulnerability and community resilience variables. Correlation testing can help describe the relationships between variable pairs, the strength of their linear association and the statistical significance [79]. Difference of means tests (independent sample *t*-tests) assessed the statistical difference of the means between the same variable but for the two independent groups of urban and rural counties [80].

We employed Spatial Scan Statistic (SaTScan) version 9.7 (SaTScan, Boston, MA, USA) for space–time cluster analyses of COVID-19 cases and fatalities across the contiguous US with Poisson and space–time prospective/retrospective models [13,23,28,29]. The removal of Hawai’i and Alaska was necessary for the space–time analysis due to contiguity requirements and coordinates for cases/fatalities were assigned based on the centroids of counties. The methodology for identifying clusters follows the SaTScan method under Poisson assumptions [81]. The space–time scan statistic is measured by a cylindrical window (with a circular geographic base and a height that reflects the time period of potential clusters), which is moved in space and time with different sizes to cover the study region and return the significant clusters. The adopted space–time model compared the values of cases/fatalities to an expected value if the spatial and temporal locations were independent [81] and the discrete Poisson model assumed that cases/fatalities follow a Poisson distribution based on the underlying county population. Both prospective and retrospective analyses are performed using space–time discrete Poisson models. In the retrospective analysis, the study region is scanned for both active clusters (lasting until the end date) and historic clusters (having ceased to exist before the study period end date), while the prospective analysis only detects active clusters. SaTScan results were limited to significant cluster outputs throughout the cumulative study period in order to focus the investigation on the greatest overall risk during the first year of the pandemic. For each cluster, we reported the counties’ Relative Risk (RR), which is the estimated risk in the cluster divided by the estimated risk outside the cluster [81]. Monte Carlo testing (999 simulations) provided the basis for statistical significance assessments.

For the remaining geospatial analyses, we utilized Geographically Weighted Regression (GWR) in ArcMap 10.8.1 (ESRI, Redlands, CA, USA) to determine the associations between independent and dependent variables, while accounting for spatial heterogeneity using geographical weights [82]. Bivariate Moran’s I tests were also performed to identify associations between two geographic variables as a measure of spatial autocorrelation [83]. Spatial lag regression modeling then examined all independent variables to determine their explanatory influence on the dependent variable (i.e., standardized cases/fatalities), which uncovers whether statistically significant spatial interactions exist directly and the strength of that interaction for each variable [84]. The GeoDA 1.18 program (Center for Spatial Data Science, University of Chicago, Chicago, IL, USA) was used for running the local bivariate Moran’s I tests, set for 999 permutations (*p*-value of 0.05 for global Moran’s I) and for the spatial lag, with a first-order queen contiguity weight assigned. Island counties (e.g., Nanucket, MA) without neighbors were included in the analyses and assigned a weight of 0 in spatial lag regression, while SaTScan cluster analyses assumed contiguity of these counties and performed the analyses as if they were connected to the mainland state. Case/fatality rates for US counties and SaTScan cluster data visualizations were created using ArcMap 10.8.1 and ArcPro 2.7 (ESRI, Redlands, CA, USA).

## 4. Results

Over 25 million confirmed COVID-19 cases and roughly 437,000 fatalities occurred in the US during our year-long study period. The results, organized by our research questions, begin with the spatial and temporal patterns and clusters, followed by the correlates of cases and fatalities and end with the geographic patterns of pre-emergent recovery.

### 4.1. Spatial and Temporal Patterns

The national trend in total cases and fatalities for the study period showed an expected periodicity by epi week. The first peak in cases occurred in April 2020, with secondary and tertiary peaks in July and December 2020. The highest newly recorded cases (1.7 million) arose in the first epi week of January 2021. The highest peak in newly recorded fatalities (23,142) also occurred in January 2021, during epi week 2.

**Figure 1:**
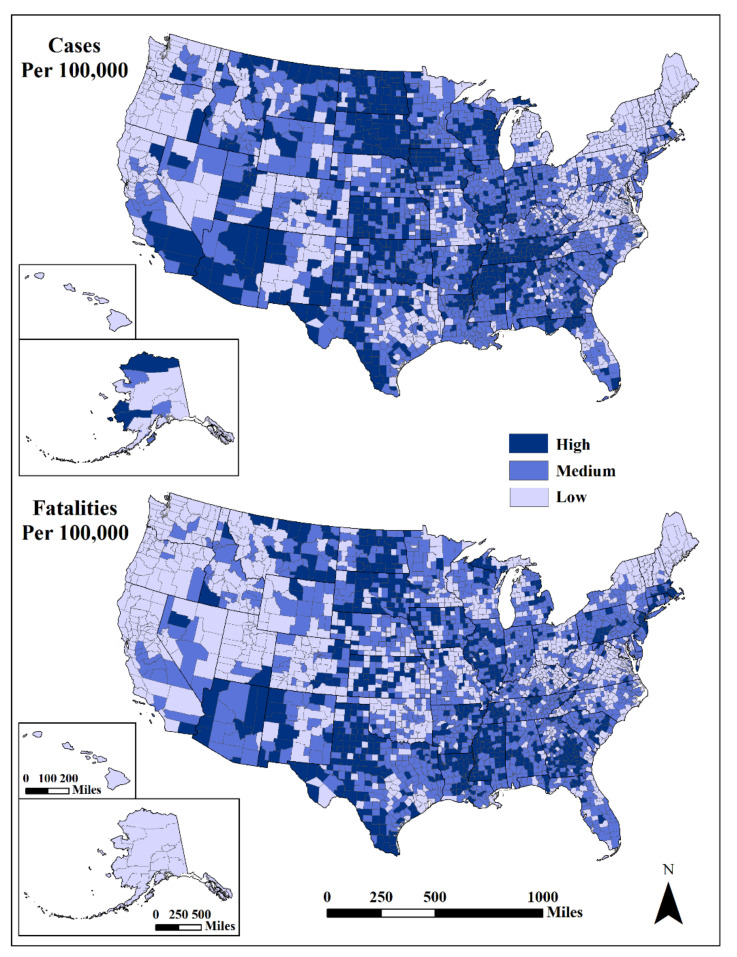
Cases (**top**) and fatalities (**bottom**) per 100,000 population. Mapped by standard deviation from the mean (high, >0.50 std. dev.; medium, from −0.50 to 0.50 std. dev.; low, <−0.50 std. dev.) using USCB [85] State and County Boundaries.

Geographically, cumulative standardized cases were highest (>9786/100,000) in the Southwest (centered on Arizona), the Great Plains/Mississippi River Valley (particularly the Dakotas) and in the South (centered on Tennessee) (Figure 1). Lower case rates (<6878/100,000) were in northern New England, New York, outside of metropolitan areas, West Virginia and coastal Washington, Oregon and northern California. The geographic distribution of high standardized fatalities (>99/100,000) had a similar pattern as cases, with less spatial density. The lowest fatalities (<51/100,000) were in northern New England, portions of Virginia and West Virginia and most western states.

#### Spatio-Temporal Clustering

Using weekly intervals, both retrospective and prospective analyses detected similar clusters. For the upper bound limit, a circular window scanning for a maximum of 20% of the total at-risk population provided a more localized clustering (county scale) of cases/fatalities. 

**Figure 2:**
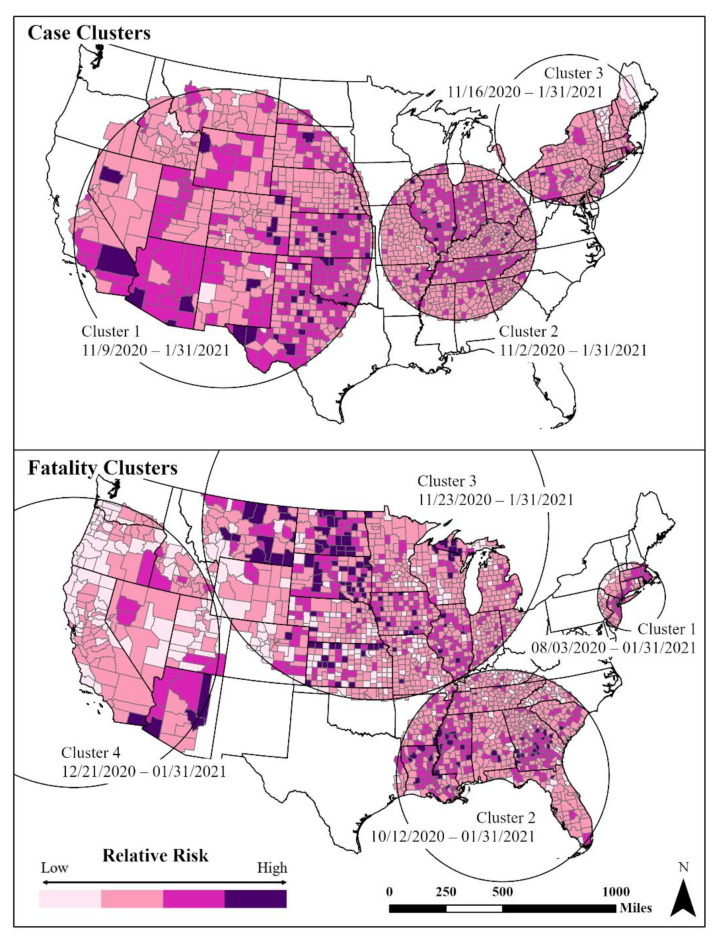
Space–time clusters of cases (low, 0.45–1.0; medium low, 1.0–3.0; medium high, 3.0–5; high, 5.0–15.39) and fatalities (low, 0–1; medium low, 1.0–3.0; medium high; 3.0–5.0; high, 5.0–21.32) using USCB [85] State and County Boundaries.

The SaTScan analysis showed three distinct and significant (*p* < 0.001) space–time case clusters from November 2020 to 31 January 2021 (Figure 2). The earliest cluster (Cluster 2) centered in the Midwest and mid-South region started on 2 November 2020 and continued to the end of our study period. Higher relative risk levels were in Tennessee, Indiana and Illinois. Cluster 1, the largest areal cluster of cases, stretched across half of the country from the Great Plains to the western states. This cluster (9 November 2020–31 January 2021) contained most of the counties with the highest relative case levels (Figure 2). The third cluster was in the northeast and covered the period from 16 November 2020 to 31 January 2021.

Four significant fatality clusters appeared, one starting in August 2020 and three in late 2020–early 2021. The earliest fatality cluster (Cluster 1) was in the northeast Megalopolis (3 August 2020–31 January 2021) (Figure 2). Within Cluster 1, there was considerable spatial variation among the counties with the highest fatality rates located in the metropolitan areas of Boston and New York–Newark. Cluster 2 (12 October 2020–31 January 2021) occurred in the Deep South, where the spatial variation shows higher fatality rates among rural counties. Fatality rate Cluster 3 (23 November 2020–31 January 2021) was centered in the northern Midwest and Great Plains regions, with a mix of both urban and rural counties. Similarly, Cluster 4 (21 December 2020–31 January 2021) in the West shows the highest fatalities in sparsely populated counties and tribal lands.

### 4.2. Case and Fatality Correlates

Results for our second research question helped explain the relationships between standardized cases and fatalities and this study’s correlates. As expected, standardized cases and fatalities were moderately correlated with one another (Table 2). However, there were significant differences between urban and rural counties in standardized cases, fatalities and their correlates (Table 2 and Table 3). While the correlation of cases and fatalities with location (rural/urban) was statistically significant, the association was relatively weak (r_s_ = −0.122 for cases and r_s_ = −0.146 for fatalities, where a positive coefficient denotes urban, a negative one denotes rural).

The pre-existing social vulnerability of counties positively correlated with standardized cases and moderately positively correlated with standardized fatalities. SoVI^®^ was strongly correlated with rural counties (r_s_ = −0.554, *p* < 0.001). The difference of means (independent samples) tests confirmed significant differences in social vulnerability between rural and urban counties, with urban counties having lower levels of social vulnerability than rural ones. In contrast, there was no consequential association between the existing levels of community resilience and standardized cases or fatalities, despite positive associations with cases (r_s_ = 0.101, *p* < 0.001) and negative correlations with fatalities (r_s_ = −0.013, *p* = 0.452). The number of total governmental mitigation measures was negatively and significantly correlated with standardized cases (r_s_ = −0.339, *p* < 0.001) and with standardized fatalities (r_s_ = −0.199, *p* < 0.001). While governmental mitigation was mildly significantly correlated with urban counties (r_s_ = 0.263, *p* < 0.001), there was a significant statistical difference between urban and rural counties in terms of the number of mitigation measures employed to reduce cases, with more measures undertaken in urban areas. 

The GWR tests did not show any significant associations between tested variables of cases and fatalities with location, total mitigation score, SoVI^®^ and BRIC. A further test of the significance of the predictors of COVID-19 cases and fatalities employed spatial lag regression models based on Lagrange multiplier tests and residuals to predict (1) standardized cases using location, total mitigation score, SoVI^®^ and BRIC as independent variables and (2) standardized fatalities using the same predictors plus cases. The model prediction of standardized cases was moderate (R^2^ = 0.434, *p* < 0.001), with higher resilience scores (β = 1420.21, *p* < 0.001) showing the most contribution, followed by lower numbers of mitigation actions undertaken (β = −238.94, *p* < 0.001), higher levels of social vulnerability (β = 95.14, *p* < 0.001) and urban locations (β = 26.38, *p* < 0.001). The model for standardized fatalities also performed moderately well (R^2^ = 0.404, *p* < 0.001) with four significant predictors, including higher resilience (β = 34.94, *p* < 0.001), higher levels of social vulnerability (β = 7.95, *p* < 0.001), mitigation actions (β = 2.81, *p* < 0.05) and standardized cases (β = 0.01, *p* < 0.001).

#### 4.2.1. Local Spatial Clusters of Cases and Fatalities 

Bivariate mapping was used to identify clusters of high cases/fatalities (hot spots), clusters of low cases/fatalities (cold spots) and spatial outliers. The Global Moran’s I test within the entire study area showed significant (*p* < 0.05) spatial association for all bivariate pairs, except fatalities and social vulnerability. The association between location and cases and fatalities for the entire study was random. The local Moran’s I test, however, showed considerable spatial clustering of cases and fatalities with social vulnerability and community resilience (all local clusters/outliers are significant, *p* < 0.05). 

**Figure 3:**
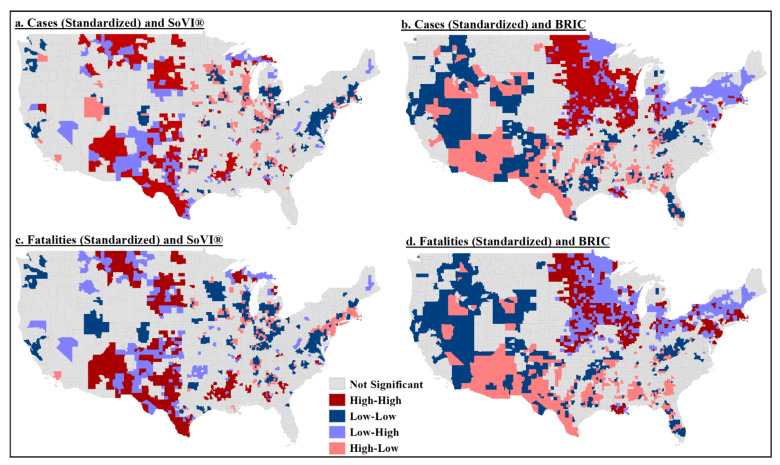
Bivariate local Moran’s I test results: (**a**) standardized cases and social vulnerability; (**b**) standardized cases and community resilience; (**c**) standardized fatalities and social vulnerability; (**d**) standardized fatalities and community resilience.

The bivariate local Moran’s I test indicated a rather significant cluster of high cases and high fatalities with high levels of social vulnerability in southern Texas, New Mexico, western Mississippi and South Dakota (Figure 3a,c). A cluster of high cases and high resilience was in the northern Plains and western Midwest counties (Iowa, Nebraska and the Dakotas) (Figure 3b). However, the most significant clustering of high fatalities and high levels of community resilience was much smaller and concentrated in upper Midwest counties (Figure 3d). There was higher statistical significance with the discordant pairs—high fatalities and low resilience and low fatalities and high resilience.

#### 4.2.2. Mitigation Clusters

Our second research question also addressed the relationship between mitigation actions and cases/fatalities and relationship variability based on rural- or urban-designated counties. There was a negative correlation between mitigation and cases/fatalities (Table 2), indicating that the more overall mitigation actions in counties, the lower cases and fatalities rates. In looking at the differences in mitigation actions themselves, we found slight but significant differences between urban and rural counties, with urban counties having undertaken more actions than rural counties (urban: x = 4.98, SD = 1.141; rural: x = 4.31, SD = 1.176).

**Figure 4:**
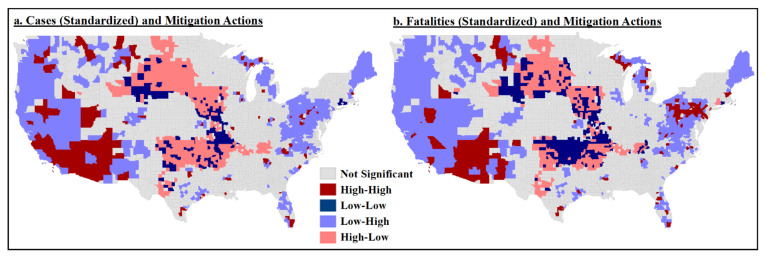
Bivariate local Moran’s I test results: (**a**) standardized cases and total mitigation actions; (**b**) standardized fatalities and total mitigation actions.

The spatial patterns of standardized cases and fatalities in relation to mitigation action levels were similar across US counties (Figure 4a,b). Counties with more mitigation actions and lower cases/fatalities were generally in the West and the East, whereas higher cases and fatalities with fewer mitigation actions were dominant in the Great Plains states. There were clusters of high cases and more mitigation actions in the Southwest, with smaller clusters in California, Montana and south Florida.

### 4.3. Pre-Emergent Recovery Spatiality and Drivers

Our third research question addressed whether there were geographic variations in unemployment change and how this related to COVID-19 cases and fatalities. Change in the unemployment rate was our proxy outcome measure to monitor pre-emergent recovery. A larger percentage increase in unemployment from 2019 to 2020 suggested a slower initial recovery.

**Figure 5:**
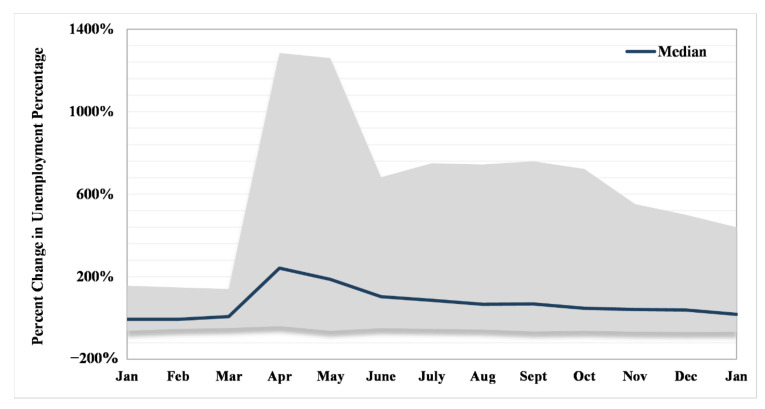
Range (gray area) and median of monthly percent change in unemployment percentage (baseline is 2019, i.e., the difference between 2020–2019 and 2021–2019).

Counties ranged in total unemployment percent change between −33.67% in Blaine County, Nebraska, and 586.31% in Maui County, Hawai’i. Temporally, the greatest range in percent change in the unemployment rate occurred in April–May 2020 with a precipitous drop in June, where it plateaued until October and then began slightly narrowing (Figure 5) in range until January 2021. The seemingly stable status (at the end of our study period) was still more than the difference between the unemployment rate in the earlier months of January and February 2020 (i.e., before business closures). A median line for counties’ unemployment percentage change showed a peak in April and a declining slope afterward, as the recovery process began.

**Figure 6:**
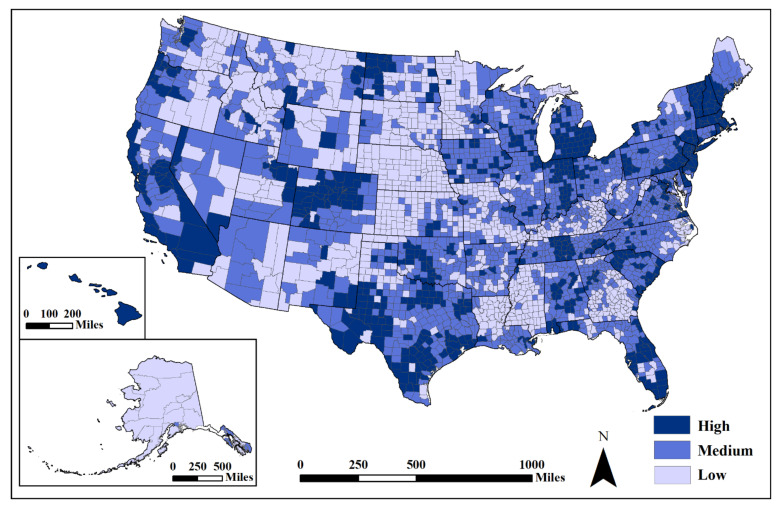
Unemployment rate percent change from 2019 to 2020. Mapped by standard deviation (high, >0.50 std. dev.; medium, from −0.50 to 0.50 std. dev.; low, <−0.50 std. dev.) from the mean (x = 0.75) using USCB [85] State and County Boundaries. Darker hues show higher percent change (more people unemployed in 2020 than 2019).

The change in unemployment from 2019 to 2020 was negatively correlated with standardized cases (r_s_ = −0.105, *p* < 0.001) and fatalities (r_s_ = −0.084, *p* < 0.001), but the strength of these associations was very weak (Table 1). As the percentage of unemployment increased from the previous year, the number of COVID-19 cases decreased slightly. The changes in unemployment were moderately associated with urban areas (r_s_ = 0.381, *p* < 0.001) and the difference of means test confirmed a statistically significant difference between urban and rural areas (Table 2). A snapshot of the status of pre-emergent recovery at the end of our study period (January 2021) shows an uneven pattern across the US, with greater increases in unemployment (less recovery) in urban counties (shown in dark hues), when compared to rural ones (shown in lighter hues) (Figure 6). Regionally, unemployment changes were more stable in some areas in the South, Great Plains and the western US, outside of the major metropolitan areas and tourist destinations.

Employing a spatial lag model with percent change in the unemployment rate as the dependent variable and cases, fatalities, location (dummy variable with rural = 0, urban = 1), total mitigation score, SoVI^®^ and BRIC generated an R^2^ = 0.511 (*p* < 0.001). The most important predictors of pre-emergent recovery during the pandemic first year were urban locations (β = 8.34, *p* < 0.001), more mitigation methods (β = 3.68, *p* < 0.001) and lower levels of pre-existing social vulnerability (β = −1.59, *p* < 0.001). 

**Figure 7:**
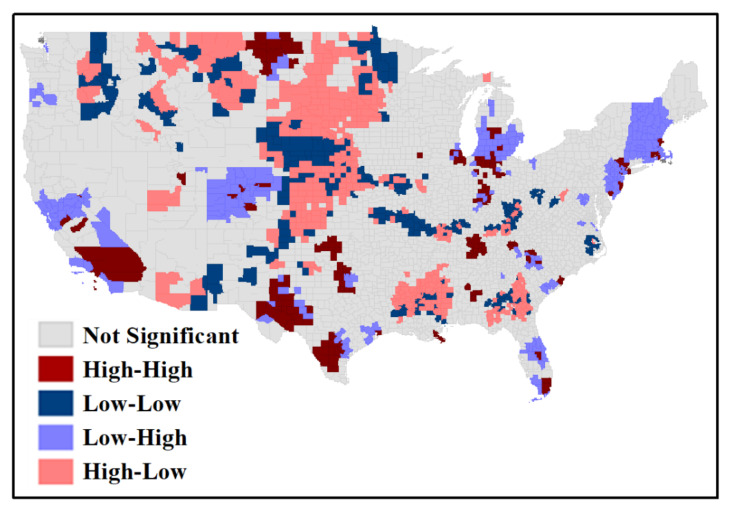
Bivariate local Moran’s I result for standardized cases and unemployment change.

The local association between cases and percent unemployment in 2020 was more significant in central US counties, where both low–low clusters and high–low outliers suggested a relatively lower unemployment change (Figure 7). However, parts of the West (e.g., California, Texas, Colorado, North Dakota and Montana), Michigan, Tennessee, South Carolina, Florida and the Northeast (New Jersey through Maine) contained high–high clusters with adjacent low–high county outliers, indicating higher rates of unemployment change.

## 5. Discussion

Investigating the spatial and temporal disparities in the first year of the COVID-19 pandemic revealed significant regional clustering patterns across US counties that varied between the annual and weekly scales. While disparate COVID-19 cumulative experiences were initially evident in the Great Plains, Southwestern and Southern regions, these patterns changed when examining the data by epi week. Space–time analyses found three distinct clusters of cases in the West/Southwest, Ohio–Mississippi Valley and Northeast from November 2020 to January 2021. Results of retrospective and prospective analysis spotted identical clusters due to the significant increase in cases at the end of 2020. Four distinct space–time clusters for fatalities showed an early cluster in the New York metropolitan region, a second cluster in the South, a third cluster in the Midwest and Great Plains and the fourth cluster in the West. In comparison with studies using the same methodology albeit focused on earlier stages of the pandemic, the identified clustering patterns are slightly different in the central and western US but rather similar in the northeastern and southern US [23,28,29]. 

Relatively few studies to date systematically examined nationwide urban–rural differences in COVID-19 cases and fatalities over the entire first year of the pandemic. The relationship between COVID-19 cases and fatalities and the correlates in the study exposes distinct urban–rural differences in US counties—more standardized cases and fatalities in rural counties than in urban counties. There were higher numbers of mitigation measures in urban areas, which also contained lower rates of standardized cases and fatalities. Our study confirms previous research investigated over a smaller geographic area that found increased standardized cases and fatalities in rural US counties [13], but contradicts a nationwide study executed earlier in the pandemic finding higher mortality rates in urban counties [30]. 

The explanatory influence of pre-existing social vulnerability, community resilience and mitigation actions correlating with COVID-19 cases/fatalities was clear in this study. In general, as the level of social vulnerability increased within a county, so did cases and fatalities. Social vulnerability was more associated with fatalities than cases, while community resilience had a less significant influence on cases or fatalities. Governmental mitigation actions had a significant association with lower cases and fatalities per 100,000 population, indicating that, as restrictions increased, cases and fatalities decreased. Spatial lag regression patterns of cases and fatalities then revealed resilience levels to be the most influential indicator, followed by social vulnerability and mitigation actions. The spatial lag results revealing higher fatality rates to be associated with higher resilience levels and increased mitigation actions seem to be a product of the Great Plains states’ experience. This area holds some of the highest levels of community resilience in the country, but high resilience does not always equate to less risk, especially in a relatively rural region with a concentration of high exposure workplaces and populations (e.g., meatpacking and immigrant labor). Additionally, the usage of one mitigation measure for the entire study period may be partially responsible for the relationship uncovered between high fatality rates and more mitigation actions. Furthermore, the relatively rural counties within this region, which are found to implement fewer mitigation actions than their urban counterparts, could be influencing the spatial lag predictors. The relationship between COVID-19 cases and fatalities and the correlates in this study exposes distinct place-based disparities among US communities not previously uncovered, particularly the strength of applying a social vulnerability or community resilience indicator. This study also supports previous research that found disadvantaged and socially vulnerable populations suffered larger, disproportionate burdens from exposure to COVID-19 [11,22]. 

Previous unobserved differential spatial patterns of unemployment change as a proxy of pre-emergent recovery were uncovered in this analysis, with higher observed changes in portions of the West, Midwest, Southeast and Northeast. Analyses show that the unemployment change rate had a stronger relationship with urbanity, adoption of mitigation actions and lower levels of social vulnerability, while it had an insignificant relationship with cases and fatalities. Reduced public interaction due to job loss partially explained the relationship between decreased COVID-19 cases and increased unemployment. Many counties with the highest rates of unemployment change were associated with tourism-based economies (e.g., Hawai’i), or densely populated urban areas, where there are more people employed in jobs that were lost due to mitigation policies. The general median trend-line for unemployment change rate showed an improvement (i.e., initiated recovery); however, there are significant disparities between counties with a wider range of difference in the unemployment rate in comparison to the pre-event situation. 

The results of this analysis provide empirical evidence for COVID-19 spatial and temporal disparities within the United States context, but they also possess international-level applicability. Countries across the globe can apply this research methodology to uncover local spatial and temporal clustering of COVID-19 cases and fatalities, test the explanatory relationship of correlate factors using the best available data and gain a better understanding of international-level unemployment recovery. Each country’s COVID-19 experience was guided by their existing healthcare system, political decisions to mitigate and local population dynamics. An increased understanding of social determinants of COVID-19 and socially vulnerable population’s experiences during the pandemic outside the United States is important for equitable recovery. As this research shows, other nations must consider multiple social and environmental factors when attempting to explain the spatial and temporal diffusion of COVID-19 cases and fatalities.

### 5.1. Limitations

Several limitations exist in this study, primarily relating to the geospatial data. Inconsistencies in COVID-19 reporting and testing led to imperfect case and fatality data, due to issues such as testing shortages and unknown county identifiers of patients. COVID-19 data may also not be an exact match to official state or county totals, due to differences in reporting between government agencies and The New York Times data collection methods. The dataset applied here also contained partially missing data for three counties in Alaska and Hawai’i, as well as the removal of those states completely for spatial analyses, but our results are no less important. Another important drawback of this investigation relates to the way SaTScan generates clusters in a circular shape, which may be limiting, due to the possibility of spatial heterogeneity within a study area. However, SaTScan’s Poisson model is based on a circular scanning window that still provides valuable spatial clustering information that help address our research questions. Our unit of analysis, US county level, may also pose a limitation due to the modifiable areal unit problem (MAUP), which can lead to changes in analysis results based on imposed geographic boundaries. A similar limitation exists due to the uncertain geographic context problem (UGCoP), which can emerge from uncertainty in the spatio-temporal contextual influence of area-based attributes on individual decision-making behaviors or outcomes [86]. Analyses at the census tract or zip code level, for example, may reveal different localized spatial patterns. However, county-level data were the only spatial scale available for US COVID-19 cases and fatalities and county level spatial units are commonly used in geographic and public health analyses. A final notable limitation is that of our proxy indicator of pre-emergent recovery, since the event (i.e., pandemic) is not over yet and the traditional definition of recovery does not match what is used here, thus our use of the term pre-emergent recovery. Furthermore, the unemployment percent change only highlights one aspect of recovery measurement and a holistic view on recovery requires additional indicators and a wider timeframe extending to the post-pandemic era.

### 5.2. Future Research Directions

Future research directions based on the results of this study could first look deeper at the dynamics of COVID-19 spatial diffusion at a more refined urban–rural categorical scale, rather than applying a binary urban versus rural schema. Other pandemic indicators relating to mitigation, such as access and receipt of vaccinations, could provide interesting explorations within and among counties of varying social vulnerability and community resilience levels, as well as political leanings, as they relate to vaccination mitigation. As more time passes since the start of the pandemic, further data have also been collected and made publicly available regarding the socio-demographic characteristics of COVID-19 patients that could also allow for expansion of our empirical understanding of spatio-temporal impacts on socially vulnerable populations.

## 6. Conclusions

To the best of our knowledge, this is one of the first studies to assess the spatial disparities of COVID-19 cases and fatalities, identify the influence of social vulnerability, community resiliency and government mitigation actions on standardized cases/fatalities across all US counties during the initial year of the pandemic. Additionally, few geographic studies have assessed the spatial variability of unemployment change rate as a measure of pre-emergent recovery, which is a crucial element directly related to the economic impact of the pandemic. Case and fatality spatial clustering found different clustering patterns than previous spatio-temporal studies executed earlier in the pandemic. Another key takeaway is the confirmation of certain urban–rural patterns observed early in the pandemic, proving those patterns of exposures and outcomes remained consistent through January 2021. More importantly, the results of this study are important for identifying place-based differences in COVID-19 exposure and outcomes based on community contextual factors and their practical application in targeting pandemic recovery at the local level. Finally, this unique longitudinal methodology applied publicly available and/or repeatable data that can guide future studies considering additional correlates for COVID-19 recovery not only in the US, but also internationally.

## Figures and Tables

**Table 1:** Input variables and sources for county-level analyses.

Variable	Time Frame	Source
COVID-19 cases and fatalities	21 January 2020–30 January 2021	The New York Times GitHUB [61]
Population	2019	ACS 5-year population estimates [62]
Social Vulnerability Index (SoVI^®^)	2018	HVRI [63]
Baseline Resilience Indicators for Communities (BRIC)	2015	HVRI [64]
County mask mandates	7 April 2020–21 July 2020	U of Chicago [65]
County emergency declarations, stay-at-home policies, business closures	All data collected 15 April 2020	National Association of Counties [66]
County business closures, mask mandates, stay-at-home orders	Last updated 2 May 2021	US Department of Health and Human Services [67]
State-level emergency declarations, shelter-in-place orders, business closures, mask mandates	Last updated 22 April 2021	Raifman et al. [68]
Economic recovery–unemployment %	January 2019–January 2021	US Bureau of Labor Statistics [69]
Urban–rural county designations	2013	NCHS [70] and USDA [71]

**Table 2:** Spearman rho correlation summary.

	Standardized Cases	Standardized Fatalities	Location (Urban/ Rural)	Statewide Mitigation	County Mitigation	Total Mitigation	Unemployment % Change	SoVI^®^	BRIC
Standardized Cases		0.502 **	−0.122 **	−0.249 **	−0.276 **	−0.339 **	−0.105 **	0.084 **	0.101 **
Standardized Fatalities	0.502 **		−0.146 **	−0.084 **	−0.201 **	−0.199 **	−0.084 **	0.251 **	−0.013
Location (urban/rural)	−0.122 **	−0.146 **		0.165 **	0.226 **	0.263 **	0.381 **	−0.554 **	0.244 **
Statewide Mitigation	−0.249 **	−0.084 **	0.165 **		0.160 **	0.580 **	0.112 **	−0.120 **	0.082 **
County Mitigation	−0.276 **	−0.201 **	0.226 **	0.160 **		0.885 **	0.214 **	−0.136 **	−0.059 **
Total Mitigation	−0.339 **	−0.199 **	0.263 **	0.580 **	0.885 **		0.242 **	−0.169 **	−0.012
Unemployment % Change	−0.105 **	−0.084 **	0.381 **	0.112 **	0.214 **	0.242 **		−0.383 **	0.143 **
SoVI^®^	0.084 **	0.251 **	−0.554 **	−0.120 **	−0.136 **	−0.169 **	−0.383 **		−0.451 **
BRIC	0.101 **	−0.013	0.244 **	0.082 **	−0.059 **	−0.012	0.143 **	−0.451 **	

** *p* < 0.01 (2-tailed).

**Table 3:** Difference of means between rural and urban counties (rural *n* = 1974, urban *n* = 1166).

Variable	Location	Mean	Standard Deviation	*t*-Value	Sig. (2-Tailed)
Standardized Cases	Rural Urban	8579.263 7913.487	3109.101 2477.920	6.604	<0.001
Standardized Fatalities	Rural Urban	164.679 129.093	114.323 71.630	10.719	<0.001
Social Vulnerability	Rural Urban	1.052 −1.793	2.285 2.204	34.155	<0.001
Community Resilience	Rural Urban	2.703 2.775	0.151 0.127	−14.190	<0.001
Mitigation	Rural Urban	4.310 4.980	1.176 1.141	−15.625	<0.001
Unemployment % Change	Rural Urban *	63.511 94.707	44.635 45.418	−18.794	<0.001

* Unemployment data unavailable in Kalawao County, Hawai’i.

## Data Availability

The dataset is published and available for download on Zenodo at http://doi.org/10.5281/zenodo.4894500 (Published on 2 June 2021).
